# Noise-driven cell differentiation and the emergence of spatiotemporal patterns

**DOI:** 10.1371/journal.pone.0232060

**Published:** 2020-04-24

**Authors:** Hadiseh Safdari, Ata Kalirad, Cristian Picioreanu, Rouzbeh Tusserkani, Bahram Goliaei, Mehdi Sadeghi

**Affiliations:** 1 School of Biological Science, Institute for Research in Fundamental Sciences (IPM), Tehran, Iran; 2 Department of Biotechnology, Faculty of Applied Sciences, Delft University of Technology, Delft, The Netherlands; 3 School of Computer Science, Institute for Research in Fundamental Sciences (IPM), Tehran, Iran; 4 Institute of Biochemistry and Biophysics, University of Tehran, Tehran, Iran; 5 National Institute of Genetic Engineering and Biotechnology (NIGEB), Tehran, Iran; Brunel University, UNITED KINGDOM

## Abstract

The emergence of phenotypic diversity in a population of cells and their arrangement in space and time is one of the most fascinating features of living systems. In fact, understanding multicellularity is unthinkable without explaining the proximate and the ultimate causes of cell differentiation in time and space. Simpler forms of cell differentiation can be found in unicellular organisms, such as bacterial biofilm, where reversible cell differentiation results in phenotypically diverse populations. In this manuscript, we attempt to start with the simple case of reversible nongenetic phenotypic to construct a model of differentiation and pattern formation. Our model, which we refer to as noise-driven differentiation (NDD) model, is an attempt to consider the prevalence of noise in biological systems, alongside what is known about genetic switches and signaling, to create a simple model which generates spatiotemporal patterns from bottom-up. Our simulations indicate that the presence of noise in cells can lead to reversible differentiation and the addition of signaling can create spatiotemporal pattern.

## Introduction

The traditional idea of a living cell where every organelle, every reaction, and every interaction is part of a clock-like order has long been shattered by the understanding that biological systems usually struggle to function in noisy environments. One might consider life to be an uphill battle against pandemonium, where disarray is the norm and spheres of order—i.e., biological systems—are rarities that are unlikely to appear in the first place. In this view, noise is a nuisance that natural selection always attempts to eliminate. It is for the same reason that selection cannot increase the fidelity of replication beyond a certain threshold; the biological cost of increasing fidelity simply becomes too high at that point [1].

A different view has recently gained some grounds [2–5]. In this view, biological systems that regulate and utilize the noise can have higher fitness under certain circumstances. Had biological systems been utterly deterministic, adaptation—i.e., the emergence of a new phenotype or a change in the gene expression pattern to utilize a new food source—would have been impossible without the emergence of new mutations. In reality, noise in the cell can result in beneficial non-genetic diversity in otherwise genetically homogenous populations—e.g., cyanobacteria [6] and yeast [7]. But what mechanism can account for the presence of phenotypic diversity amongst daughter cells that are genetic clones of each other? Is it possible for a stochastic mechanism to explain the non-genetic diversity? Even if such stochastic explanation were offered, how could this explanation possibly account for the ordered spatiotemporal patterns in spatially-extended cell population?

The model of cell differentiation proposed in this work, henceforth referred to as the noise-driven differentiation (NDD) model, accounts for the peculiarities of this biological phenomenon by weaving noise into an explanation of cellular behaviors at the time of differentiation. While on the surface, this approach might seem lofty and even radical, the model discussed in this paper is parsimonious when it comes to the mechanisms requisite for its operation. The NDD model rests on 8 components (Table 1). Some can be regarded as facts, based on reliable empirical evidence from biological systems (components #1 and #2), while others are more accurately described as assumptions (components #3–8).

**Table 1:** The components of the NDD model.

Components		Justifications
1	Noise, resulting from a plentitude of sources, is an inseparable part of a living cell.	Based on the observed effect of noise on the processes in living cells, from microbes to mice (e.g., see [8–12]).
2	Stochastic partitioning of cytoplasm during cell division and the random distribution of molecules in the cytoplasm determine the cytoplasmic contents of the daughter cells.	Variation in the position of cell-division plane is a biological fact (reviewed in [13–17]), and its effect on the diversification of cells is well-known (e.g., see [18, 19]).
3	The fate of a cell is determined when it is born.	Based on the assumption that cell-fate-determining factors are in small numbers in a cell and the stochastic distribution of these factors during cell division determines the fate of the newly-born daughter cells.
4	Cell fate is determined by a switch.	Genetic switches have been observed in a variety of taxa (reviewed in [2]), and has been proposed as a model to account for cell differentiation (e.g., see [20]).
5	The interaction between the building blocks of the switch determines its bias.	Our assumption based on our knowledge of well-known genetic switches, such as λ phage (see [21]).
6	All the information needed to construct the switch is genetic.	We assume that, while stochasticity is what drives the decision made by the switch, the information necessary to construct the switch is encoded in the genetic content of a cell.
7	The robustness of the switch is the result of a complex network of interactions.	Our assumption based on [22].
8	Cell fate is influenced by its location and its environment.*	We assume the the switch determining cell fate should, in addition to being swayed by the intrinsic factors, be influenced by its neighbors.

* This component is necessary for the ordered spatiotemporal patterns in a population of cells.

There is a plethora of phenomena within a cell that can contribute to its intrinsic noise—e.g., transcription regulation, transcription factor binding to the DNA, RNA processing in eukaryotes, translation, post-translational modifications, protein complex formation, protein and RNA degradation, etc. Single-cell level measurements of gene expression further cements the notion that cells are intrinsically noisy when it comes translating its genotype into phenotype [23]. The displacement of the division plane relative to the middle of the cell can result in an unequal distribution of cell content between the daughter cells, even if molecules are homogeneously distributed within the cell. In fact, the central role of asymmetric cell division in the diversification of cells, from *Drosophila* to mammals has been known for many years [18, 19]. The components #1–2 reflect the role of stochasticity in living systems based on these observation.

Thus far, two types of solutions to the problem of cell differentiation have been proposed: the first category consists of models that rely on cell-cell communication (reviewed in [24]) and the second category relies on asymmetric cell division (reviewed in [25]). The research project within the confines of the former category is mainly a quest to find the building blocks of the apparatus that makes the specific kind of cell-cell communication needed for cell differentiation. The latter category, on the other hand, presumes the asymmetric cell division to result in differentiation. Hitherto unknown and often complicated mechanisms have been proposed to explain the asymmetric distribution of fate-determining factors during cell division [26, 27]. Both categories rely on physical interactions at the cellular level. While we agree with the importance of the asymmetric cell division, it seems to us that a stochastic model of differentiation, like the NDD model, negates the need for new mechanisms. In this model, we adopt the view that stochastic processes result in differentiated cells due to the distribution of key proteins, instead of cells differentiating by receiving signals after they are born (component #3).

The component #4 is based on the idea that characteristics of a cell can be changed by a switch (Not a very recent idea, e.g., [28]). The notion that cell fate is determined by a switch is best illustrated by the now famous case of the λ phage. The process by which the phage decides to integrate into the host’s genome—i.e., lysogenic—or to replicate copies of itself in the cell until it bursts open—i.e., lytic—can be explained by a stochastic switch which makes that portentous decision in a probabilistic fashion, while taking into account the presence of certain key factors [29]. One can assume that the bias of this switch is determined by the interactions of its building blocks (component #5). For example, upon infecting bacterial cells, λ phage proceeds to lyse the host, but as the concentration of CII protein increases, so does the likelihood of the reactions suppressing the activation of *pR* and *pL* promoters, relevant to the onset of the lytic trajectory, which in turn, tilts the scale away from lysis towards lysogeny [21]. We propose that phenotypic diversity arises from the effect of the noise on a genetic circuit that exhibits a switch-like behavior (component #6). The notion that different phenotypes are produced from the same genotype as a consequence of noise is widely observed in nature (reviewed in [30]).

How robust can be a fate-determining toggle switch in the face of new mutations? Sharifi-Zarchi [22] took advantage of the gene expression profiles of 442 mouse embryonic cells to construct a network of key transcription factors (TFs). While a regulatory circuit with two TFs could explain differentiation, They reasoned that such a simple switch is susceptible to mutations. To construct a robust switch, they built a circuit with two clusters of TFs with correlated expressions. Expectedly, the alternative switch, which involved more interactions, was much more robust. We would expect different levels of robustness for a switch, given its biological importance in evolution (component #7).

**Fig 1:**
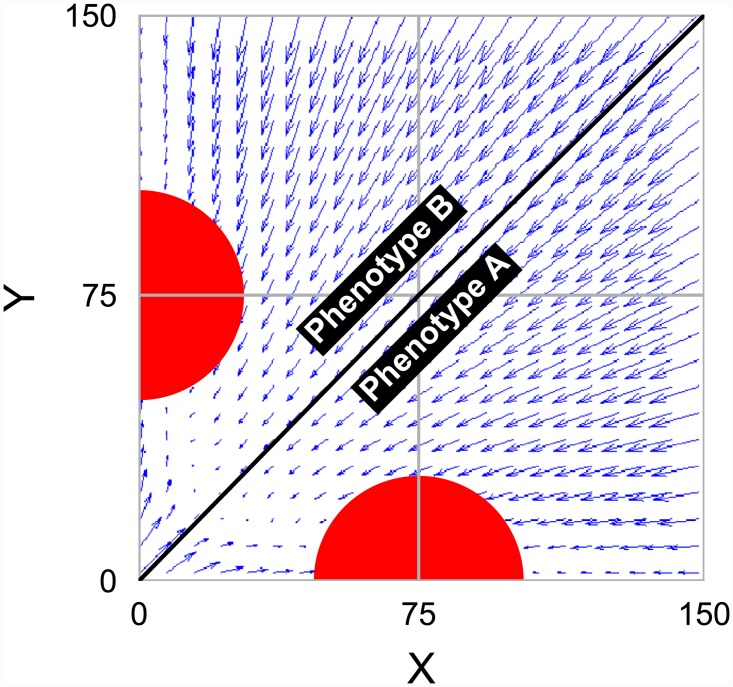
The phase-portrait diagram for the NDD model (based on Eq 1). In a bistable switch, two attractors (red semicircles) and, consequently, two phenotypes are available: *A* and *B*. The likelihood of a switch choosing state *A* over *B* depends on the number of the transcription factor associated with state *A* (TF_*X*_) relative to the number of the transcription factor associated with state *B* (TF_*Y*_), as well as the noise in its environment. The parameters used to generate this and the following figure are as follows: *n* = 2, *β* = 0.1, protein half-life = 10min, and protein dissociation constant = 10. Unless noted otherwise, these parameters are used in all the subsequent figures.

The components #1–7 are sufficient to generate a population of cells with different proportions of two phenotypes (Fig 1). While this kind of fate determination is adequate vis-à-vis primitive cells with no organization, it does not allow the emergence of multicellularity. An additional component is necessary to explain this major transition from mere phenotypic differentiation to ordered spatiotemporal patterns in the body of a multicellular organism. For self-organization to occur, we assume that the toggle switch determining cell fate should, in addition to being swayed by the intrinsic factors, be influenced by its neighbors (component #8).

To test the general veracity of the NDD model, we used a simple model of cell aggregation. In this model, a simple switch is defined that can switch between phenotypes, *A* and *B*. We look at the noise resulting from the random distribution of fate-determining proteins during cell division (component #1), the stochastic positioning of cytoplasmic cell division (component #2), and the effect of signaling from neighboring cells on other cells (component #8) on a model of cell aggregation where the fate of cell depends on a genetic switch (components #4–7). In the discussion, we juxtapose the NDD model with some of the more recent attempts at modeling cell differentiation.

## Materials and methods

In the cell aggregation model, the population is made up of cells, where each cell is a circular particle defined by its state variables—e.g., spatial position, size, and phenotype. The simulation geometry is a *L* × *L* square and no flux boundaries. It is assumed that the relative amount of two key transcription factors, *X* and *Y*, controls the cell types; hence, in this model, a cell can have two phenotypes, *A* and *B*, as shown in Fig 1. The dominance of protein *X* leads to phenotype *A* and the dominance of protein *Y* results in phenotype *B*. In fact, a positive feedback loop influences the decision-making process. Two negatively coupled repressors mutually inhibit the expression of the gene that encodes the other repressor- i.e., a toggle switch (component #4). The rate of this mutual repression is represented in the form of a Hill function [31]. This positive feedback loop results in two stable steady states, hence implies non-linear dynamical equations. Non-linear differential equations govern the changes in the number of the repressor proteins, *X* and *Y* (Fig 1);

$$\begin{array}{cc} & \frac{dX}{dt}=\frac{\beta }{1+Y^{n}}-X\;,\\ & \frac{dY}{dt}=\frac{\beta }{1+X^{n}}-Y\;. \end{array}$$ (1)

**Fig 2:**
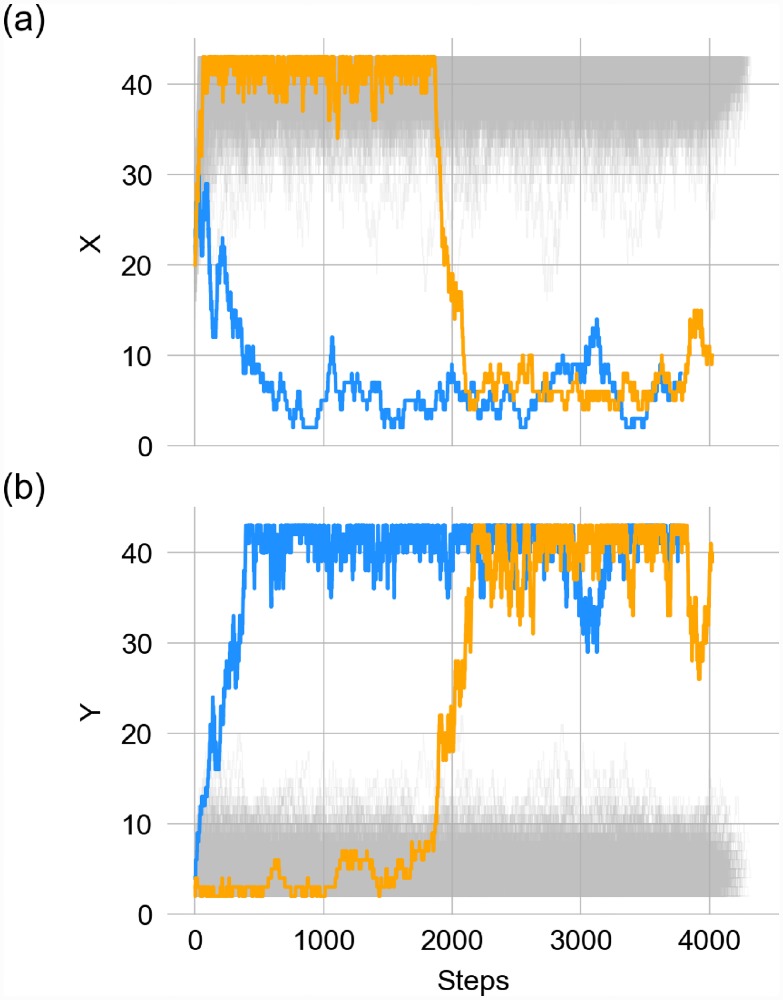
As cells grow, they stochastically explore the phase-plane around their attractor (as depicted in Fig 1)—I.e., over time the values for transcription factors *X* (a) and *Y* (b) for each cell fluctuate around the attractor that was determined when the cell was born. These fluctuations can result in a cell moving away from its original attractor towards the other attractor, such that it will be more likely for its daughters to have phenotypes different from their parent. The trajectories follow the TFs counts during their lifespan. The blue and orange lines represent the trajectories with a transition from one attractor to another one; on the contrary, grey trajectories indicates small fluctuation of TFs counts around their attractor without a transition. Results are based on 512 cells that descended from a single cell in the cell aggregation model.

Here, *β* is the effective rate of protein synthesis and *n* is the Hill coefficient, which represents the degree of competence. The number of repressors are represented in the unit of their dissociation constants, *K*_*s*_, and time is rescaled by degradation rate of proteins [31–33]. Biologically-reasonable values were chosen for the parameters used in our simulation such that Eq 1 would be bistable (following [31]). This bistable regulatory network has two attractors corresponding to its stable steady states. Based on the amount of proteins at the cell division time, the cell can be in the domain of each attractor, which determines its fate. Depending on the intensity of inhibitory effects of TFs (through the values of constants in the Hill function [31]), the two domains of attractors could be equal or not (component #5). Fig 2 shows an example of such behavior in our cell aggregation model. S1 Movie shows the changes in the distribution of TFs in cells around their attractors during the emergence of generation 12.

### Population growth algorithm

Simulation starts with a single cell with phenotype A. Each iteration in the simulation can be divided into four steps:

1. *Cell growth*: The repressor proteins inside the cytoplasm interact with each other and their numbers, *X* and *Y*, are updated; however, because of their low copy numbers, instead of deterministic equations (Eq 1), their fluctuations are captured by the Gillespie algorithm [34] as a stochastic dynamics for discrete values. According to this algorithm, a probability of occurrence will be assigned to every biochemical reaction in the system. Every protein (*X* or *Y*) is produced with a probability according to the first term on the right hand sides of the Eq 1. As the number of protein *X* increases, it further represses the production of protein *Y* and vice versa. Every protein degrades proportional to its number. In every step of the Gillespie algorithm, one of the above reactions occurs and the time will be updated. The process continues until the number of proteins reaches a steady state. The production of *X* and *Y*, until it reaches a steady state, is simulated as a “burst”, independent of cell growth. After this burst of gene expression, cells grow linearly in size.
2. *Cell division*: Even after the number of proteins in a cell reaches the steady state, the cell continues to grow. The growth stops only after the cell reaches a critical size. At this point the cell divides into two daughter cells. The content of the mother cell is distributed among her daughters according to a uniform distribution. In reality and in the presence of active transportation, one can still expect a uniform distribution of molecules in the cytoplasm [35], making this assumption biologically reasonable. The position at which cell division occurs is randomly chosen based on a normal distribution, $N(0,\sigma^{2}),$ which its mean value is the mid plane of the cell (also the reference point), and *σ*^2^ as the variance around the mean value (component #2). In this study *σ*^2^ varies up to 0.1 parentage of the radius of the cell. At the time of birth, the phenotype of each newborn cell is determined based on the cytoplasmic contents (number of key proteins, *X*, and *Y* at the time of birth) inherited from the mother cell (component #3). During the cell growth, the number of each protein has a stochastic trajectory in the domain of its attractor and finally it will reach its steady state. In this model, phenotypic change is reversible, meaning that the phenotype can change between the two possible states over generations. Since in our simulations, daughter cells have similar volumes, we consider the number of proteins distributed between them, and not their concentrations.
3. *Relaxation*: After a cell divides, the cells push each other outwards to make room for the new daughter cells [36]. Simulation proceeds by repeating the steps #1-3. It is worth noting that, without considering self-organization, the process described above would result in a disordered blob of cells.
4. *Self-organization*: To involve the self-organization phenomenon in the process of cell maturation (component #8), cells secrete some signaling molecules, with concentration *C*_*s*_, which affects the propensities in the Gillespie algorithm and, consequently, the production of proteins. The signaling molecules diffuse in the medium according to the following reaction-diffusion equation:
  $$\begin{array}{c} \frac{\partial C_{s}}{\partial t}=D_{s}\nabla^{2}C_{s}+k_{sp}C_{B}-k_{sc}\frac{C_{s}}{K_{s}+C_{s}}\left(C_{A}+C_{B}\right)-k_{sd}C_{s}\;. \end{array}$$ (2)
  Here, *k*_*sp*_, *k*_*sc*_ and *k*_*sd*_ represent, respectively, the rate of production, consumption and decay of the signaling molecules and *D*_*s*_ is the diffusion coefficient of the signaling molecules. *C*_*A*_ and *C*_*B*_ respectively show the number of cells with phenotype *A* and *B* at each point of the medium. In our simulations, we used *D*_*s*_ = 10^−11^ *m*^2^/*s*, *k*_*sp*_ = 0.01*kg*^−1^ *s*^−1^, *k*_*sc*_ = 0.0001*kg*^−1^ *s*^−1^, *k*_*sd*_ = 0.01*s*^−1^, and the protein dissociation constant is *K*_*s*_ = 0.01*m*^−3^.
  In these simulations, the secreting cells are those with phenotype *B*; hence, the production of signaling molecules is proportional to the amount of B cells. Since both phenotypes consume these molecules, the consumption depends on the number of both A and B cells. When *B* cells emerge, they secret signaling molecules, which diffuse in their environment. The effective concentration of the signaling molecules at any location determines if a cell at that location is affected by the signal, which would decrease the production of protein *X* and augment the production of protein *Y*. Consequently, their surrounding cells would have less chance of producing protein *X* and their offspring is less likely to be in the domain of attraction of protein *X*.

## Results

The overall behavior of the cell aggregation model demonstrates the principles of our framework—that is, the stochasticity results in phenotypic heterogeneity as the population grows in size (S1 Movie). To further illustrate how each source of noise affects the cell differentiation, we focused on each source separately in the simulations.

### The stochastic positioning of division plane and the stochastic distribution of key proteins affect differentiation

One source of intrinsic stochasticity stems from the random positioning of the division plane. This factor would disproportionately influence the number of molecules that exist in low numbers within cytoplasm. In this work, it has been postulated that the determinants of cell fate are low in numbers and thus, greatly affected by stochasticity.

**Fig 3:**
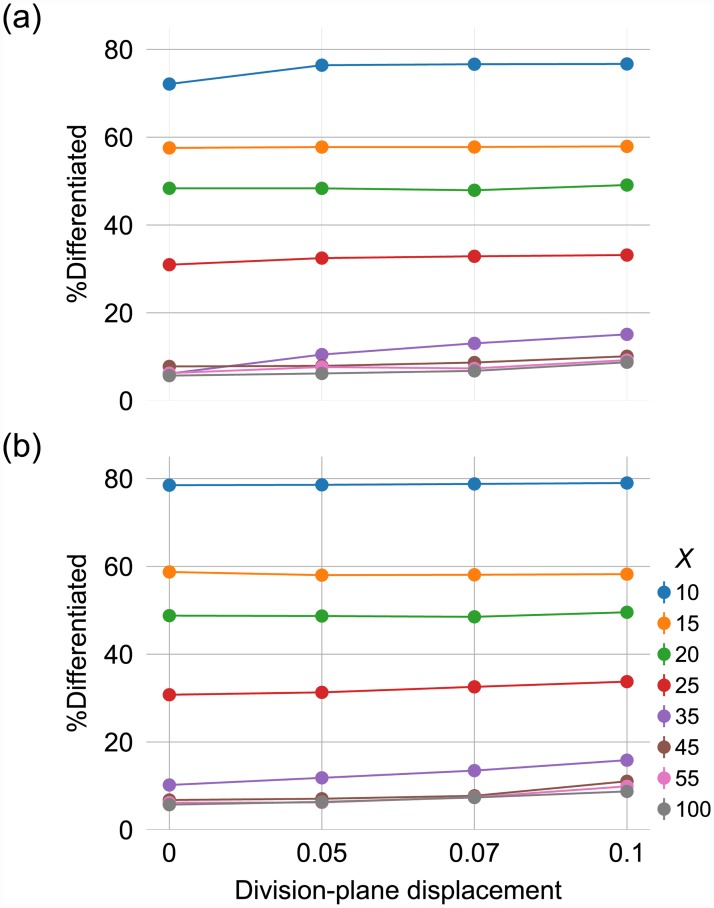
The stochastic positioning of the division plane and the random distribution of TFs in the cytoplasm, as intrinsic sources of noise, affect the non-genetic phenotypic diversity (component #2). The phenotypic diversity is represented by the proportion of cells with the phenotype *B* relative to the total number of cells in the population. In panel (a), the only source of noise is the stochastic positioning of the division plane (*σ*^2^ = 0.1), while panel (b) shows the phenotypic diversity as a result of both variance in division-plane (*σ*^2^ = 0.1) and the noise resulting from drawing the number of TFs at any location along the cell at random from a uniform distribution. In each panel, the curves indicate different amounts of protein *X* in the mother cell. The results are average over 100 replications. Error bars are 95%CI. The error bars are too small to be seen.

To demonstrate this phenomenon, the position of the division plane was allowed to vary with respect to the mid plane of the cell. Starting from a cell with phenotype *A*, in which the protein *X* is dominant, the population heterogeneity –i.e., emergence of phenotype *B*– was traced over 12 generations. The results are shown in Fig 3. When the division plane is situated in the middle of the cell, and the TFs are relatively abundant, very few cells differentiate. As the variance in the cell-division plane—i.e., *σ*^2^ in the normal distribution used to choose the position of division plane—increases, so does the proportion of *B* cells. This phenomenon is dependent on the number of proteins, since such a bias is more pronounced when the number of proteins is relatively low. In fact, with large numbers of TFs in a cell, it will be more likely for its daughters to have almost the same density of TFs as their mother. Thus, they will be in the same domain as the mother in the phase space, and their fates will be identical to hers. This can be seen clearly in the lower curves in Fig 3. However, for low copy numbers of TFs, the difference between TF numbers in two daughter cells becomes more prominent and can even lead to different cell fates. Therefore, it is possible to have heterogeneity in the population in the absence of any other noise, i.e., cells with low TF numbers are heterogeneous even with no variance in division-plane displacement (Fig 3a). In Fig 3a, the distribution of TFs is deterministic across the length of the cell—e.g., if *X* = 10 and cell divides exactly on its equator, each daughter receives 5 TFs. We add stochasticity to the distribution of TFs by randomly drawing the number of TFs at any location along the length of the cell from a uniform distribution (*U*(0, *L*), where *L* is the length of the cell, provided $\sum_{l=0}^{L}X_{l}=TF_{X}\frac{U(0,L)}{\left||U(0,L)|\right|}$ and $\sum_{l=0}^{L}Y_{l}=TF_{Y}\frac{U(0,L)}{\left||U(0,L)|\right|}$). This additional source of noise has a tangible, though slight, effect on the percentage of differentiated cell (Fig 3b).

### Signaling can create spatial order

**Fig 4:**
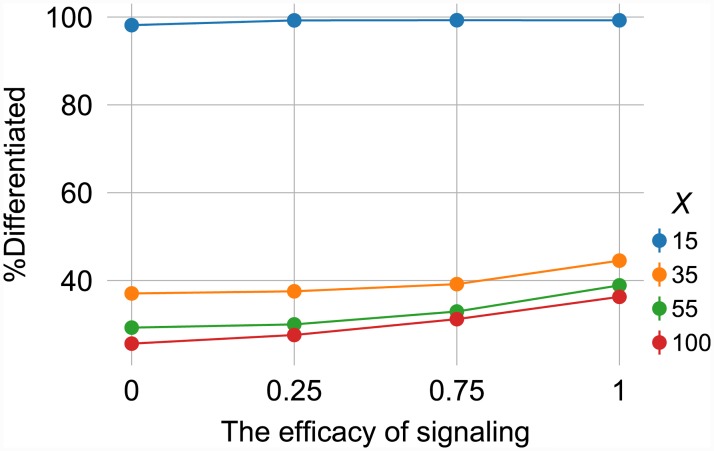
Adding signaling to the cell aggregation model results in higher none-genetic phenotypic diversity, compared to populations without signaling (as shown in Fig 5). The phenotypic diversity is represented by the proportion of cells with the phenotype *B* relative to the total number of cells in the population. The curves indicate different amounts of protein *X* in the mother cell. It fascinating to notice how the lowest number of TFs (*X* = 10) results in total differentiation. The efficacy of signaling is defined as follows: if in the position of a cell with phenotype *A*, the signal concentration exceeds the mean signal concentration, then this cell would have more chance of becoming a *B* cell. The results are average over 100 replications. Error bars are 95%CI. The error bars are too small to be seen.

In the cell aggregation model, *B* cell can release signals in the environment. These signals diffuse at a slow rate and, consequently, have a very short radius of influence. The absorption of these signals by other cells in the population affects the number of proteins involved in the switch—that is, switching to the phenotype *B* during cell division becomes more likely (Fig 4). When this environmental signaling is added to the population, the cells organize in a non-random fashion, a stark contrast to the random heterogeneity observed before (S2 Movie).

**Fig 5:**
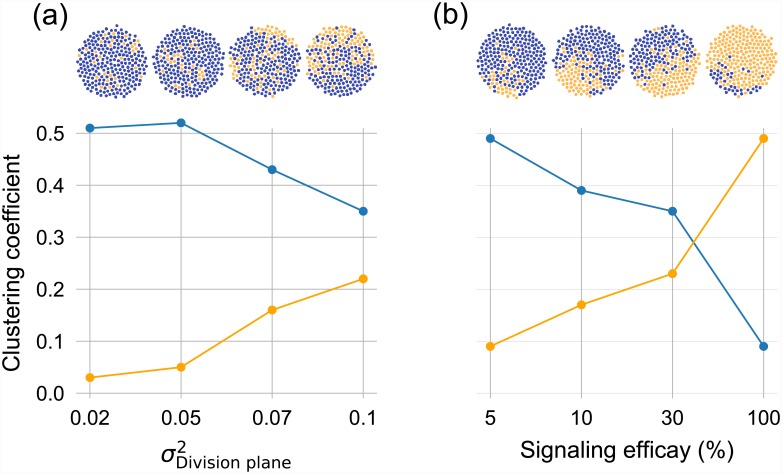
Upper panels: Population heterogeneity as a result of increase in: (a) the noise of the stochastic positioning of the division plane of cells (increase in the variance of the Normal distribution) or (b) the intensity of the secreted signals from the *B* cells. Both the number of TFs in (a) and the efficacy of signaling in (b) increases from left to right in this figure. The blue circles represent the *A* cells and the orange ones represent the *B* cells. Each aggregation is the final state of a single run of the stochastic model after 8 generations with the given parameters, starting from a blue cell. The amount of protein *X* in the initial cell in each simulation was 35. The radius of the area of aggregation is 100*μm*. Bottom panels: The change in the global clustering coefficient of both cell types as a result of change in the (a) noise of the stochastic positioning of the division plane of cells and (b) intensity of the signaling molecules secreted by orange cells. In the latter case, the variance of the noise in the division plane of cells is fixed to the 0.05 of the cells radios. The blue curves stands for A cells and the orange curves for B cells.

Fig 5 represents a visual understanding of the results from the NDD model. It shows the bacterial community in a 2-dimensional simulation area after 8 generations. In Fig 5a, the variance in the stochastic positioning of the division plane increases from left to right. It can be seen that the heterogeneity in the population increases as well by the presence of new phenotypes (cells in orange). In Fig 5b, development of an organized community as a result of signaling molecules is apparent (group of orange cells). The curves in the lower plots show the change in the global clustering coefficient of two cell types A and B, as the increase in the intensity of noise. In graph theory, the clustering coefficient, *C*, represents a measure of cohesion of a graph, i.e., how clustered vertices are, $C=\frac{3(\#\;\text{triangles}\;\text{on}\;\text{the}\;\text{graph})}{\left(\#\;\text{connected}\;\text{triples}\;\text{of}\;\text{vertices}\right)}$ [37, 38]; here a triangle means three neighbour cells. It could be seen that in the presence of signaling, Fig 5b, the clustering coefficient of orange cells increases strongly; which means the differentiated cells created a denser population. The organization observed will increase over time and the community of orange cells will develop (S3 Movie).

## Discussion

Molecular processes in the cell are noisy events that result in varying degrees of heterogeneity. Taming this inherent noise is vital for the emergence and the continuation of life. In fact, life can be characterized as a system with the capacity to control noise. The phenotype of a cell is generally stable, but during cell division, this cell can produce daughter cells with different phenotypes via symmetric or asymmetric cell division. The resulting non-genetic phenotypic diversity is a way to achieve adaptation in a fluctuating environment by producing phenotypically diverse offspring without any need for genetic change. Given the variety of sources of noise, the cell fate determination can be a stochastic process. One can imagine a few genes involved in cell fate determination, where the noise in the cell affects the proportion of daughter cells born with a certain phenotype. The ability to change the phenotypic proportion of daughter cells via a stochastic mechanism, which is also tunable, is a superb strategy to outcompete rivals bereft of such gift.

Proposing a stochastic model of cell differentiation is not an entirely novel concept, e.g., see [39, 40] as examples of an impressive body of work produced by Kunihiko Kaneko and his colleagues on this subject and [41, 42] as similar proposals regarding the possible role of stochasticity in generating phenotypic diversity. The NDD model is an attempt to merge observations about the role of noise in living systems and the prevalence of toggle switches in biological processes to explain cell differentiation. Here, we utilized a bistable switch to demonstrate how such model would create population-level heterogeneity and how signaling can give spatial order to this heterogeneity. This approach is in keeping with the recent emphasis on the importance and the prevalence of noise in biological functions, specifically cell fate [2, 4, 43]. The model of cell aggregation used in this study allowed us to test all the components of the NDD model, barring components #6 and #7, which demand through investigations of their own. This model of cell aggregation provides us with a relatively realistic depiction of the process that results in phenotypic differentiation in a population. To illustrate the virtues of our approach, it is worth considering how it differs with some of the more prominent approaches to explain cell differentiation.

Olimpio et al. [44] explored the role of cell-cell communication in the emergence of spatial order in a cellular automaton. In their model, *N* cells exist on a lattice, and there is no cell growth or division, thus the effect of the distribution of fate-determining proteins in the mother cell on the fates of the daughters (component #3 of the NDD model) does not factor in. When they include noise in their model, it consists of a Gaussian noise added to the activation threshold of the switch. In the NDD model presented here, we consider different sources of noise affecting biological systems separately, and in this work we looked at the effects of some of these sources on the emergence of spatiotemporal heterogeneity. In the NDD model, the intrinsic noise alone generates heterogeneity without including cell signaling.

Perez-Carasco et al. [20], use a bistable genetic switch to translate the analog morphogen gradient into a spatial pattern. Similar to Olimpio et al. [44], cells do not divide and the noise, when introduced, is in the form of transcriptional bursts. The point is not to downplay the relevance of these works, far from it; the NDD model takes the idea of a toggle switch and the role of noise and attempts to (i) tease apart the different sources of noise and (ii) organize the observations and the assumptions needed to explain the emergence of spatial heterogeneity in living organisms.

The results from our cell-aggregation model shows that the NDD model can provide an explanation for the emergence of spatial heterogeneity, simply based on the intrinsic noise in living systems. Without cell-cell communication (component #8), the mere presence of noise (components #1–3), coupled with a genetic toggle switch (components #4–7), can generate spatial heterogeneity. The addition of signaling generates the spatial pattern. Our results further cement the notion that a noise-driven system can generate cell differentiation. In their criticism of a noise-driven alternative to their model, Suzuki [39] considered it unlikely for a noise-driven model to maintain the exact levels of stochasticity needed to produce the desired proportion of differentiated cells to stem cells. In our view, if a switch is robust (component #7 of the NDD model), then it will be able to maintain its bias in the face of new mutations.

It has not escaped our notice that a bistable switch, as described here, can only create reversible cell differentiation, as seen in prokaryotes. In fact, Suzuki [39] points out this problem with a model of cell differentiation based on a bistable toggle switch. Can the NDD model be used to simulate the irreversible eukaryotic differentiation from stem cell to differentiated cell? Replacing the bistable switch with a multi-stable variant might solve this problem [45]. In fact, in a follow-up work by Khorasani, Sadeghi, and Nowzari-Dalini [46], replacing the bistable switch by a tri-stable one was enough to generate irreversible differentiation.

The ability of the cells to differentiate into different types was the crucial step that enabled the ancient solitary cells to leave the primordial soup behind and evolve into the vast array of specialized cells we see today. As Queller [47] point out, there are different shades of organismality –i.e., the ability for components to work together with little conflict among them–, each shade resulting from the affinity of the members of the system to cooperate versus the temptation to cheat. We can sidestep the problem of conflict since in prokaryotic multicellularity, e.g., biofilm, and in most truly multicellular eukaryotes, the cells are highly related, thus lowering the probability of cheating [48]. Without tangible levels of conflict, multicellularity as a trait becomes patently advantageous. In their seminal work, Maynard Smith and Szathmáry [49] considered two possible mechanisms to account for the emergence of cell differentiation: one relies on the presence of determinants that prohibit the stem cell to differentiate, and the other postulates the cell-cell contact as a mechanism that determines cell fate. While these suggestions account for how the multicellularity might be sustained, they do not explain how this major evolutionary transition could have occurred in the first place.

One of the quintessential aspects of the discussed model is its population-level perspective. Population-level thinking is one of the main points of the evolutionary theory, and bringing it to explain a cellular phenomenon can lead us to reap valuable insights. While a population of cells has, on average, certain properties relevant to differentiation, e.g., the mean number of key proteins, the average position of cell division plane, and etc., these average values do not tell the whole story. Instead, the variance in these values, i.e., the non-genetic variation present amongst individuals, is the key to understand differentiation (as observed in studies such as in [12, 50]). This noise in the population is essentially the fuel that propels cellular differentiation, be it in the reversible differentiation in prokaryotes or the more complicated irreversible ones in higher organisms. We believe that this population-level vantage point is the necessary tool to understand this otherwise mind-boggling biological process. Without this perspective, the task of explaining such a seemingly fine-tuned process devolves into an attempt to come up with complex cellular interactions that would make climbing this improbable biological mountain feasible.

The fact that NDD model breaks down the components required for spatiotemporal heterogeneity, makes it easy to muse about the events that led to the different levels of multicellularity during the evolution of life on Earth. It is easier for cell differentiation to evolve via the emergence of a switch, rather than the less plausible path that involves the evolution of a clockwork mechanism. According to the NDD model, the emergence of early stages of multicellularity only requires the evolution of a suitable switch—the rest of the necessary ingredients needed for the transition into self-organization is provided by the stochastic elements affecting the switch. The major transition from unicellularity to multicellularity –i.e., from phenotypic diversity in a population to from an ordered and stable spatial heterogeneity– only requires one more step: the evolved switch should be simply affected by the signal(s) released by its neighbors (components #8). The spatial information received in this way would bias the switch such that the population-level organization is retained. The NDD model is the logical extension of earlier ideas describing the role of stochasticity in phenotypic variation and the switch-like behavior of genetic circuits vis-à-vis differentiation and multicellularity (e.g., see [51]). It is tempting to postulate a connection between the cell-differentiation switch, postulated in the NDD model, and the toggle switch used in quorum sensing in bacteria [52]. Quorum sensing enables bacteria to regulate their phenotypes apropos of their neighbors and is more robust in a dense community [53]. It seems plausible to consider this type community-based phenotypic regulation as a precursor to similar switch-based mechanisms for cell differentiation in multicellular organisms.

## Conclusions

The NDD model is an attempt to construct a model of cell differentiation which takes into account the mounting evidence concerning the role of noise in biological systems. The components of this model consists of biological observations and a few assumptions. Using a cell aggregation model, we show that these components are sufficient to generate spatiotemporal heterogeneity in population of cells. The bistable switch allows for the simulation of the reversible cell differentiation. In a followup work, we used a tri-stable switch to explore the irreversible differentiation using the NDD framework. We believe that breaking down the minimum biological components necessary for cell differentiation provides a framework to reconstruct the chain of events that were required for different levels of multicellularity to emerge during the history of life on Earth.

## Supporting information

S1 Movie The change in the distribution of TFs within cells just before they divide. Parameters used are the same as Fig 1. S2 and S3 Movies show 3-dimensional simulations of a community of cells in a layer. Simulation performed in a *L* × *L* × *h* cube and starts with one cell at the centre, the red cell with type A. There is a single layer of cells with height *h*, corresponding to the diameter of a single cell. The cells grow in volume; after reaching a critical volume they divide– the same as the two dimensional case– their cytoplasmic content distributes between the two daughter cells.
(MP4)

S2 Movie The emergence of heterogeneity in the population of cells as a result of the presence of noise in the process of cell growth and division. The average amount of TFs in each cell at steady state is 25. The simulation starts by one cell and continues over 13 generations, *L* = 130*μm* and *h* = 1.33*μm*. The population starts from a red cell of type A and at the end it contains a mixture of type A and cells with type B (orange color).
(MP4)

S3 Movie The formation of a spatial organization as a result of the secretion of signaling molecules, which diffuse in their environment and affect the differentiation of the cells. The average amount of TFs in each cell at steady state = 25. The simulation starts by one cell and continues over 13 generations, *L* = 130*μm* and *h* = 1.33*μm*. Red cells represent type A and orange cells have type B.
(MP4)

## References

[1] Kimura M. On the evolutionary adjustment of spontaneous mutation rates. Genet Res. 1967;9(1):23–34. 10.1017/S0016672300010284

[2] Balázsi G, van Oudenaarden A, Collins JJ. Cellular Decision Making and Biological Noise: From Microbes to Mammals. Cell. 2011;144(6):910–925. 10.1016/j.cell.2011.01.030 21414483 PMC3068611

[3] Chalancon G, Ravarani C, Balaji S, Alfonso M, Aravind L, Jothi R, et al Interplay between gene expression noise and regulatory network architecture. Trends Genet. 2012;28(5):221–232. 10.1016/j.tig.2012.01.006 22365642 PMC3340541

[4] Huang S. Non-genetic heterogeneity of cells in development: more than just noise. Development. 2009;136(23):3853–3862. 10.1242/dev.035139 19906852 PMC2778736

[5] Losick R, Desplan C. Stochasticity and cell fate. Science. 2008;320(5872):65–68. 10.1126/science.1147888 18388284 PMC2605794

[6] Wolk PC. Heterocyst formation. Annu Rev Genet. 1996;30:59–78. 10.1146/annurev.genet.30.1.59 8982449

[7] Paliwal S, Iglesias PA, Campbell K, Hilioti Z, Groisman A, Levchenko A. MAPK-mediated bimodal gene expression and adaptive gradient sensing in yeast. Nature. 2007;446(7131):46–51. 10.1038/nature05561 17310144

[8] Kepler TB, Elston TC. Stochasticity in Transcriptional Regulation: Origins, Consequences, and Mathematical Representations. Biophys J. 2001;81(6):3116–3136. 10.1016/S0006-3495(01)75949-8 11720979 PMC1301773

[9] Ozbudak EM, Thattai M, Kurtser I, Grossman AD, van Oudenaarden A. Regulation of noise in the expression of a single gene. Nat Genet. 2002;31(1):69–73. 10.1038/ng869 11967532

[10] Elowitz MB, Levine AJ, Siggia ED, Swain PS. Stochastic Gene Expression in a Single Cell. Science. 2002;297(5584):1183 10.1126/science.1070919 12183631

[11] Maamar H, Raj A, Dubnau D. Noise in Gene Expression Determines Cell Fate in *Bacillus subtilis*. Science. 2007;317(5837):526–529. 10.1126/science.1140818 17569828 PMC3828679

[12] Chang HH, Hemberg M, Barahona M, Ingber DE, Huang S. Transcriptome-wide noise controls lineage choice in mammalian progenitor cells. Nature. 2008;453(7194):544–547. 10.1038/nature06965 18497826 PMC5546414

[13] Margolin W. Themes and variations in prokaryotic cell division FEMS Microbiol Rev. 2000;. 10.1111/j.1574-6976.2000.tb00554.x 10978550

[14] Monahan L, Liew A, Bottomley A, Harry E. Division site positioning in bacteria: one size does not fit all. Front Microbiol. 2014;5:19 10.3389/fmicb.2014.00019 24550892 PMC3910319

[15] Pickett-Heaps JD, Gunning BE, Brown RC, Lemmon BE, Cleary AL. The cytoplast concept in dividing plant cells: cytoplasmic domains and the evolution of spatially organized cell division. Am J Bot. 1999;. 10.2307/2656933 21680355

[16] Wu J, Tzanakakis E. Contribution of Stochastic Partitioning at Human Embryonic Stem Cell Division to NANOG Heterogeneity. PLOS ONE. 2012;7(11):e50715 10.1371/journal.pone.0050715 23226362 PMC3511357

[17] Bradshaw N, Losick R. Asymmetric division triggers cell-specific gene expression through coupled capture and stabilization of a phosphatase. eLife. 2015;4:e08145 10.7554/eLife.08145 26465112 PMC4714977

[18] Jan YN, Jan LY. Asymmetric cell division. Nature. 1998;392:775 EP –. 10.1038/33854 9572136

[19] Betschinger J, Knoblich JA. Dare to Be Different: Asymmetric Cell Division in *Drosophila*, *C.elegans* and Vertebrates. Curr Biol. 2017;14(16):R674–R685. 10.1016/j.cub.2004.08.017 15324689

[20] Perez-Carrasco R, Guerrero P, Briscoe J, Page KM. Intrinsic Noise Profoundly Alters the Dynamics and Steady State of Morphogen-Controlled Bistable Genetic Switches. PLOS Comput Biol. 2016;12(10):1–23. 10.1371/journal.pcbi.1005154 PMC5074595 27768683

[21] Cortes MG, Trinh JT, Zeng L, Balázsi G. Late-Arriving Signals Contribute Less to Cell-Fate Decisions. Biophys J. 2018;113(9):2110–2120. 10.1016/j.bpj.2017.09.012 PMC5685783 29117533

[22] Sharifi-Zarchi A, Totonchi M, Khaloughi K, Karamzadeh R, Araúzo-Bravo MJ, Baharvand H, et al Increased robustness of early embryogenesis through collective decision-making by key transcription factors. BMC Syst Biol. 2015;9(1):23 10.1186/s12918-015-0169-8 26033487 PMC4450992

[23] Sanchez A, Golding I. Genetic determinants and cellular constraints in noisy gene expression. Science. 2013;342(6163):1188–1193. 10.1126/science.1242975 24311680 PMC4045091

[24] Wolpert L. Positional information and patterning revisited. J Theor Biol. 2011;269(1):359–365. 10.1016/j.jtbi.2010.10.034 21044633

[25] Rudel D, Sommer RJ. The evolution of developmental mechanisms. Dev Biol. 2003;264(1):15–37. 10.1016/S0012-1606(03)00353-1 14623229

[26] Morrison SJ, Kimble J. Asymmetric and symmetric stem-cell divisions in development and cancer. Nature. 2006;441(7097):1068–1074. 10.1038/nature04956 16810241

[27] Clevers H. Stem cells, asymmetric division and cancer. Nat Genet. 2005;37(10):1027–1028. 10.1038/ng1005-1027 16195718

[28] Novick A, Weiner M. Enzyme induction as an all-or-none phenomenon. PNAS. 1957;43(7):553–566. 10.1073/pnas.43.7.553 16590055 PMC528498

[29] Ptashne M. A Genetic Switch: Phage Lambda Revisited. Cold Spring Harbor Laboratory Press; 2004.

[30] Vogt G. Stochastic developmental variation, an epigenetic source of phenotypic diversity with far-reaching biological consequences. J Biosci. 2015;40(1):159–204. 10.1007/s12038-015-9506-8 25740150

[31] Gardner TS, Cantor CR, Collins JJ. Construction of a genetic toggle switch in *Escherichia coli*. Nature. 2000;403(1038):1183. 10.1038/35002131 10659857

[32] Carson E, Cobelli C. Modelling Methodology for Physiology and Medicine. Academic Press; 2000.

[33] Elowitz MB, Stanislas L. A synthetic oscillatory network of transcriptional regulators. Nature. 2000;403(1038):1183. 10.1038/35002125 10659856

[34] Gillespie DT. Exact stochastic simulation of coupled chemical reactions. J Phys Chem. 1977;81(25):2340–2361. 10.1021/j100540a008

[35] Huh D, Paulsson J. Random partitioning of molecules at cell division. PNAS. 2011;108(36):15004–15009. 10.1073/pnas.1013171108 21873252 PMC3169110

[36] Kreft JU, Picioreanu C, Wimpenny JWT, van Loosdrecht MCM. Individual-based modelling of biofilms. Microbiology. 2001;147(11):2897–2912. 10.1099/00221287-147-11-2897 11700341

[37] Newman MEJ. Clustering and preferential attachment in growing networks. Phys Rev E. 2001;64:025102 10.1103/PhysRevE.64.025102 11497639

[38] Watts DJ, Strogatz SH. Collective dynamics of ‘small-world’ networks. Nature. 1998;393(6684):440–442. 10.1038/30918 9623998

[39] Suzuki N, Furusawa C, Kaneko K. Oscillatory Protein Expression Dynamics Endows Stem Cells with Robust Differentiation Potential. PLOS ONE. 2011;6(11):e27232–. 10.1371/journal.pone.0027232 22073296 PMC3207845

[40] Yamagishi JF, Saito N, Kaneko K. Symbiotic Cell Differentiation and Cooperative Growth in Multicellular Aggregates. PLOS Comput Biol. 2016;12(10):1–17. 10.1371/journal.pcbi.1005042 PMC5066942 27749898

[41] Kupiec JJ. A Darwinian theory for the origin of cellular differentiation. Mol Gen Genet. 1997;255(2):201–208. 10.1007/s004380050490 9236778

[42] Paldi A. Stochastic gene expression during cell differentiation: order from disorder? Cell Mol Life Sci. 2003;60(9):1775–1778. 10.1007/s00018-003-23147-z 14523542 PMC11138758

[43] Kittisopikul M, Süel GM. Biological role of noise encoded in a genetic network motif. PNAS. 2010;107(30):13300–13305. 10.1073/pnas.1003975107 20616054 PMC2922135

[44] Olimpio EP, Dang Y, Youk H. Statistical Dynamics of Spatial-Order Formation by Communicating Cells. iScience. 2018;2:27–40. 10.1016/j.isci.2018.03.013 30428376 PMC6135931

[45] Ghaffarizadeh A, Flann N, Podgorski G. Multistable switches and their role in cellular differentiation networks. BMC Bioinformatics. 2014;15(S7):1–13. 25078021 10.1186/1471-2105-15-S7-S7 PMC4110729

[46] Khorasani N, Sadeghi M, Nowzari-Dalini A. A Computational Model of Stem Cell Molecular Mechanism to Maintain Tissue Homeostasis. bioRxiv. 2020;. 10.1371/journal.pone.0236519 PMC7392222 32730297

[47] Queller DC, Strassmann JE. Beyond society: the evolution of organismality. Philos Trans R Soc Lond B Biol Sci. 2009;364(1533):3143 10.1098/rstb.2009.0095 19805423 PMC2781869

[48] Ostrowski EA, Shaulsky G. Learning to get along despite struggling to get by. Genome Biol. 2009;10(5):218 10.1186/gb-2009-10-5-218 19519929 PMC2718508

[49] Maynard Smith J, Szathmáry E. The Major Transition in Evolution. New York: Oxford University Press; 1995.

[50] Moussy A, Cosette J, Parmentier R, da Silva C, Corre G, Richard A, et al Integrated time-lapse and single-cell transcription studies highlight the variable and dynamic nature of human hematopoietic cell fate commitment. PLOS Biology. 2017;15(7):e2001867–. 10.1371/journal.pbio.2001867 28749943 PMC5531424

[51] Nanjundiah V. Cellular Slime Mold Development as a Paradigm for the Transition from Unicellular to Multicellular Life In: Niklas KJ, Newman SA, editors. Multicellularity: Origins and Evolution. Cambridge, Massachusetts: The MIT Press; 2016 p. 105–130.

[52] Hooshangi S, Bentley WE. LsrR Quorum Sensing “Switch” Is Revealed by a Bottom-Up Approach. PLOS Comput Biol. 2011;7(9):1–11. 10.1371/journal.pcbi.1002172 PMC3182856 21980272

[53] Schluter J, Schoech AP, Foster KR, Mitri S. The Evolution of Quorum Sensing as a Mechanism to Infer Kinship. PLOS Comput Biol. 2016;12(4):1–18. 10.1371/journal.pcbi.1004848 PMC4847791 27120081

